# Effect of the Anionic Counterpart: Molybdate vs. Tungstate in Energy Storage for Pseudo-Capacitor Applications

**DOI:** 10.3390/nano11030580

**Published:** 2021-02-26

**Authors:** Pratigya Sharma, Manickam Minakshi, Jonathan Whale, Annelise Jean-Fulcrand, Georg Garnweitner

**Affiliations:** 1 Engineering and Energy, Murdoch University, Perth, WA 6150, Australia; Pratigya.Sharma@murdoch.edu.au (P.S.); j.whale@murdoch.edu.au (J.W.); 2 Institut für Partikeltechnik, Technische Universität Braunschweig, Volkmaroder Straße 5, 38104 Braunschweig, Germany; a.jean-fulcrand@tu-braunschweig.de; 3 Laboratory for Emerging Nanometrology, Technische Universität Braunschweig, Langer Kamp 6A, 38106 Braunschweig, Germany

**Keywords:** energy storage, electrode materials, asymmetric pseudo-capacitor

## Abstract

Nickel-based bimetallic oxides (BMOs) have shown significant potential in battery-type electrodes for pseudo-capacitors given their ability to facilitate redox reactions. In this work, two bimetallic oxides, NiMoO_4_ and NiWO_4_, were synthesized using a wet chemical route. The structure and electrochemical properties of the pseudo-capacitor cathode materials were characterized. NiMoO_4_ showed superior charge storage performance in comparison to NiWO_4_, exhibiting a discharge capacitance of 124 and 77 F·g^−1^, respectively. NiMoO_4_, moreover, demonstrates better capacity retention after 1000 cycles with 87.14% compared to 82.22% for NiWO_4_. The lower electrochemical performance of the latter was identified to result from the redox behavior during cycling. NiWO_4_ reacts in the alkaline solution and forms a passivation layer composed of WO_3_ on the electrode, while in contrast, the redox behavior of NiMoO_4_ is fully reversible.

## 1. Introduction

The successful transition to fully sustainable energy systems cannot be achieved without improving the management and storage of energy. Thereby, the development of a wide range of energy storage devices is key in order to satisfy all type of applications. In recent years, research studies on energy storage systems, such as fuel cells, batteries, pseudo-capacitors, and supercapacitors have been extremely active, especially for applications such as transportation and renewable energy [1].

Unlike batteries, supercapacitors possess high power density but exhibit low energy density, limiting their use. Pseudo-capacitors were developed to fill the gap between batteries and supercapacitors. They achieve a better energy density compared to supercapacitors by storing charge chemically through redox reactions. In order to improve the energy density while maintaining excellent power density and cycle stability properties, various electro-active materials are being investigated, which are able to store the energy via quick redox reactions occurring at the interface between the electrode and electrolyte. In particular, transition metal oxides such as RuO_2_, MnO_2_, NiO, and CoO are widely investigated as cathode materials for such pseudo-capacitors [2,3]. However, metal oxides possess a high electrical resistance and usually need to be combined with conductive electroactive materials like activated carbon, carbon fibers (CF), and carbon nanotubes (CNT) [4]. By doping a single metal oxide with other transition metals, a novel material with multiple oxidation states can be obtained, inducing redox reactions, in analogy to a battery electrode, and reducing the charge transfer resistance [5]. In this context, nickel-based bimetallic oxides (BMOs) are excellent candidates for battery-type electrodes for pseudo-capacitors. Several Ni-BMOs were developed, such as NiMoO_4_ [6], NiCo_2_O_4_ [5], and NiMnO [7]. A range of BMOs were previously synthesized via various routes including sol-gel synthesis [8,9], ball milling [10], and hydrothermal strategies [6,9], which resulted in highly diverse nanostructures, such as mesoporous materials [11,12], nanosheets [5], and nanowires [6]. The formation of nanostructured BMOs is an effective way to increase the specific surface area and to obtain a higher specific capacitance. More metal centers are accessible to promote redox reactions and to facilitate electron and ion exchange at the interface [2], hereby, improving the capacitative performance.

NiMoO_4_ was previously investigated for pseudo-capacitor applications because of its high electrochemical energy storage density, which originates from the high capacitance of NiO (theoretical capacitance (~2500 F/g) and the enhanced conductivity due to MoO_3_). NiMoO_4_ can be synthesized in the α-phase and the β-phase [6], and it was observed that β-NiMoO_4_ possesses a higher electrical conductivity than α-NiMoO_4_ [13].

In contrast, NiWO_4_ has been investigated predominantly as a catalyst for wastewater treatment [11]. However, its excellent electrical conductivity (~10^−7^ to 10^−3^ S·cm^−1^) compared to NiO (10^−13^ S·cm^−1^) can be beneficial for energy storage systems [14]. At the present time, NiWO_4_ has barely been studied for pseudo-capacitor applications [14,15]. Niu [14] observed that NiWO_4_ exhibits higher capacitance when in its amorphous form.

The use of engineered materials with a controlled structure and crystal properties is crucial for the improvement of energy storage systems [16]. In this study, the influence of the anion counterpart (molybdate vs. tungstate) of the synthesized Ni-based BMOs is studied in detail. NiMoO_4_ and NiWO_4_ nanostructures are synthesized using a single step low-temperature hydrothermal process. The crystal structure and morphology of the obtained products are investigated using X-ray diffraction (XRD) and electron microscopy (SEM and TEM). The molecular structure of the material is characterized with Raman and infrared spectroscopy as well as energy-dispersive X-ray spectroscopy (EDX). The influence of the type of anion on the structure is then compared to the electrochemical performance. The synthesized Ni-based BMOs have been electrochemically activated by cyclic voltammetry and tested as the electrode for a pseudo-capacitor. The nickel-based BMOs are cycled against an activated carbon anode to obtain an asymmetric pseudo-capacitor. Finally, the electrochemical properties of both Ni-based BMOs are investigated using cyclic voltammetry and the constant current-constant voltage (CC/CV) charge method. The obtained electrochemical properties are compared and related to chemical and structural differences between the two materials.

## 2. Materials and Methods

### 2.1. Synthesis of Nickel Molybdate and Nickel Tungstate

**Figure 1:**
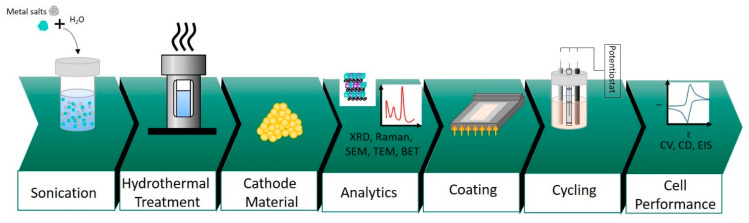
Schematic illustration of the various process steps from synthesis to characterization.

All the chemicals used were of an analytical grade, obtained from Sigma Aldrich, New South Wales, Australia, and used without further purification. A facile hydrothermal approach was adopted as a route for synthesizing NiMoO_4_ and NiWO_4_ nanoparticles [9]. In a typical synthesis, for nickel molybdate (NiMoO_4_), 1 mmol of nickel nitrate (Ni(NO_3_)_2_·6H_2_O) and 1 mmol of sodium molybdate (Na_2_MoO_4_·2H_2_O) were dissolved in deionized water under sonication conditions. The homogenous solution was then transferred to a 50-mL Teflon-lined stainless-steel autoclave, which was sealed and heated to 140 °C for 12 h to carry out the reaction. The product was then washed with a copious amount of ethanol and deionized water and, finally, dried overnight in an oven maintained at 60 °C. The final product termed as “NiMoO_4_” was then subjected to various physical and electrochemical characterizations in this study and the entire process is illustrated in Figure 1. Likewise, nickel tungstate (NiWO_4_) was synthesized under identical conditions except 1 mmol of sodium tungstate (Na_2_WO_4_·2H_2_O) was used instead of sodium molybdate. The hydrothermal synthesis of tungsten oxide (WO_3_) is described in the supplementary information section (Figure S1).

### 2.2. Physical Characterization of NiMoO_4_ and NiWO_4_

Raman spectra (Alpha300 R—Confocal Raman Imaging, with a 532 nm Nd:YAG laser, WITec, Ulm, Germany) were recorded to provide information on the chemical composition. The crystallinity and phase identification of the as-prepared powder samples was carried out using X-ray diffraction (XRD) with a Cu Kα radiation (Empyrean Cu LEF HR goniometer, Almelo, Netherlands) on a Si sample holder, operating at 40 kV and 30 mA over the 2θ range of 10°–70° (Empyrean series 2, PANalytical PIXcel-3D detector, Almelo, Netherlands).X-ray photoelectron spectra (XPS) were obtained using a Kratos AXIS Nova instrument (Kratos Analytical, Manchester, UK) with the aid of monochromatic Al-κ_α_ radiation operating at 15 kV and 10 mA. A pressure of < 1 × 10^−7^ Pa was maintained and the C 1s peak was corrected at 284.8 eV prior to analysis. The particle size distribution of the two synthesized materials was measured by a LUMiSizer device from LUM GmbH, Berlin, Germany. The samples were first dispersed in N-methyl-2-pyrrolidone (NMP) at a 5 wt.% concentration. Then the particles in suspension were sedimented at three different speeds (300 rpm for 300 s, 2000 rpm for 200 s, and 4000 rpm for 200 s). The structural and composition properties of the samples were studied using scanning electron microscopy (SEM) with a secondary electron (SE) detector (Helios G4 CX Dual-Beam, Thermo Fisher Scientific, MA, USA) as well as transmission electron microscopy (TEM) (Tecnai G2 F20, FEI, USA). The surface area was evaluated by nitrogen sorption with the Brunauer–Emmett–Teller (BET) method (NOVA 2000e, Quantachrome, Odelzhausen, Germany).

### 2.3. Electrochemical Analysis

To investigate the differences in the electrochemical properties between molybdate and tungstate, electrochemical measurements were carried out in 2 M NaOH solution using a BioLogic SP-150 workstation (BioLogic Science Instrument, Seyssinet-Pariset, France). For three-electrode configurations, either the molybdate or tungstate sample, served as a working electrode. A platinum wire (1 mm in diameter and 10 cm in length) was used as a counter electrode, and mercury-mercuric oxide (Hg/HgO) served as a reference electrode. The working electrodes were composed of NiMoO_4_ or NiWO_4_, carbon black, and poly-vinylidene fluoride (PVdF) in the weight ratio of 75:15:10, respectively. The materials were dispersed in 400 µL of N-methyl-2-pyrrolidone (NMP) and mixed in an agate mortar, producing a slurry. The homogenous mixture (approximately 7 mg for both NiMoO_4_ and NiWO_4_) was then coated (as shown in Figure 1) on a graphite sheet as a current collector (having an area of 1 cm^2^), and was subsequently dried in an oven at 80 °C for 5 h. Cyclic voltammetry (CV) and charge-discharge experiments were measured in a potential window of 0 to 0.6 V.

For a two-electrode configuration, the synthesized materials (NiMoO_4_ or NiWO_4_) were used as a positive electrode, and activated carbon (AC) as the negative electrode. To test the suitability of the cathode material for asymmetric pseudo-capacitor applications, the measurements were carried out within the potential range of 0–1.6 V. Electrochemical impedance spectroscopy (EIS) was performed in a frequency range from 0.01 to 100,000 Hz using an open circuit potential. The specific capacitance (*C_s_*), energy density (*E*), and power density (*P*) of the asymmetric pseudo-capacitor were calculated using the following relations.

(1) $$C_{s}\;=\;\frac{i\Delta t}{m\Delta V}$$

(2) $$E\;=\;\frac{1}{2}C_{s}V^{2}$$

(3) P = 3600E/Δt

where *i* is the constant discharge current (A), Δ*t* is the discharge time (s), m is the mass of active material (g), and Δ*V* is the potential window (*V*).

The mass ratio of the positive and negative electrodes was balanced using the equation below for an asymmetric device, to aid in sustaining the charge conservation between two electrodes.

(4) $$\frac{m^{+}}{m^{-}}\;=\;\frac{C_{s-}\times \;\Delta V_{-}}{C_{s+}\times \;\Delta V_{+}}$$

In the above equations, m represents the mass of active material in g. $C_{s-}$, $C_{s+}$, and $\Delta V_{-}$, $\Delta V_{+}$ represent the specific capacitance and potential window of the negative and the positive electrode, respectively. For a three-electrode configuration, the specific capacitance of NiMoO_4_ and NiWO_4_ was calculated to be 392.53 F·g^−1^ and 246.25 F·g^−1^, respectively. Using Equation (4), an optimized mass ratio between anode and cathode material was determined. The mass ratio between NiMoO_4_ and activated carbon (AC) was calculated to be 0.917, whereas that of NiWO_4_ and AC was 1.461. For 15 mg of NiMoO_4_, 13.75 mg of AC was taken whereas, for 15 mg of NiWO_4_, 21.91 mg of AC was taken and, hence, the asymmetric capacitor was constructed.

## 3. Results and Discussion

### 3.1. Chemical and Structural Characterization of NiMoO_4_ and NiWO_4_

**Figure 2:**
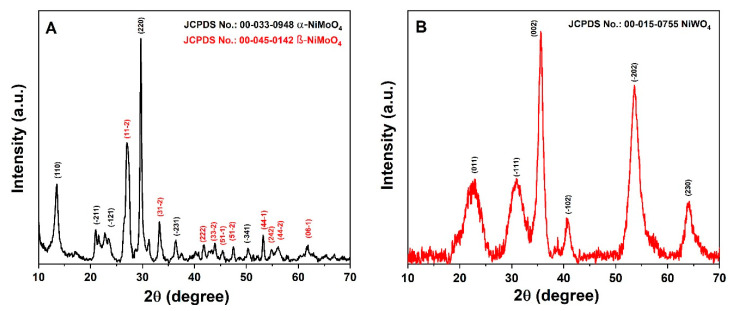
X-ray diffraction patterns of hydrothermally synthesized (**A**) α- and β-NiMoO_4_, and (**B**) NiWO_4_ as indicated in the figure.

**Figure 3:**
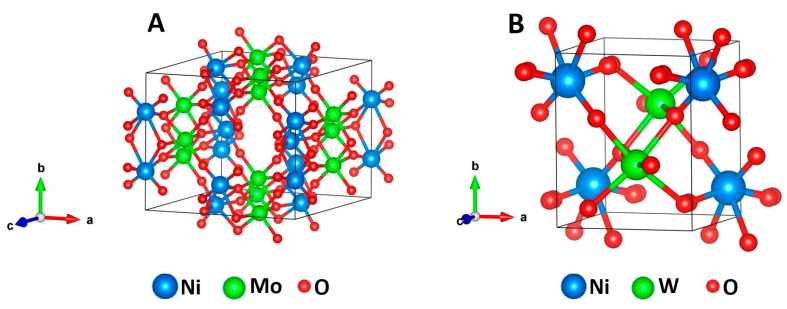
Crystal structure of monoclinic (**A**) α-NiMoO_4_ and (**B**) NiWO_4_ illustrating the coordination relationship of NiO_6_ with MoO_6_ octahedra and distorted WO_6_ in NiMoO_4_ and NiWO_4_, respectively.

Figure 2A,B show the powder X-ray powder diffraction (XRD) patterns of the synthesized products. It was observed that, at given identical synthesis conditions, the pattern of the NiMoO_4_ sample (Figure 2A) shows sharp reflections and, hence, is highly crystalline. The main reflections observed in the given 2*θ* range are at the values of 14.29, 21.73, 23.96, 28.82, 36.41, and 50.64° and can be correlated with the (110), (-211), (-121), (220), (-231), and (-341) reflections of the monoclinic α-phase of NiMoO_4_ (standard JCPDS card no. 00-033-0948), with lattice parameters a = 9.50 Å, b = 8.75 Å, and c = 7.66 Å, β = 113.13° and a space group of I2/m [6]. In addition, further reflections at 2*θ* positions of 27.19, 33.69, 41.74, 43.87, 45.57, 47.59, 53.58, 54.59, 56.13, and 61.74 ° were assigned to the (11-2), (31-2), (222), (33-2), (51-1), (51-2), (44-1), (242), (44-2), and (06-1) planes, respectively, corresponding to the β-phase of NiMoO_4_ (standard JCPDS card no. 00-045-0142). The lattice parameters for this β-phase are a = 10.18 Å, b = 9.24 Å, c = 7.02 Å, and β = 107.09° with a space group of C2/m [6]. Hence, the presence of both the α-form and the β-form of NiMoO_4_ is observed in the XRD analysis, while the α-phase is the predominant one. On the other hand, in Figure 2B, much broader reflections are observed for the product of the nickel tungstate synthesis, indicating small crystallite sizes and lower crystallinity. The individual signals at 23.96, 30.92, 36.57, 41.66, 54.62, and 63.70° 2*θ* correlate well with the (011), (-111), (002), (-102), (-202), and (230) reflections of wolframite-type NiWO_4_ (standard JCPDS card no. 00-015-755) with the lattice parameters a = 4.60 Å, b = 5.66 Å, and c = 4.91 Å, β = 90.01°, and a space group of P2/c. The standard monoclinic structure of α-NiMoO_4_ and NiWO_4_ is illustrated in Figure 3. In the α-NiMoO_4_ crystal structure (Figure 3A), the Mo sites possess a six-fold coordination geometry with O atoms. In the case of β-NiMoO_4_ (not shown), two types of Mo-O coordination can be observed, which are (a) Mo-O possessing an octahedral coordination, similar to the one observed for α-NiMoO_4_, and (b) a tetrahedral coordination with Mo atoms at the centers forming irregular tetrahedra. The Ni atom is coordinated with six O atoms forming edge-sharing NiO_6_ octahedra in both the α-phase and the β-phase. On the other hand, NiWO_4_ (Figure 3B) features W sites coordinated to six O atoms forming distorted WO_6_ octahedra, which share corners with eight surrounding NiO_6_ octahedra and edges with two neighboring WO_6_ octahedra.

**Figure 4:**
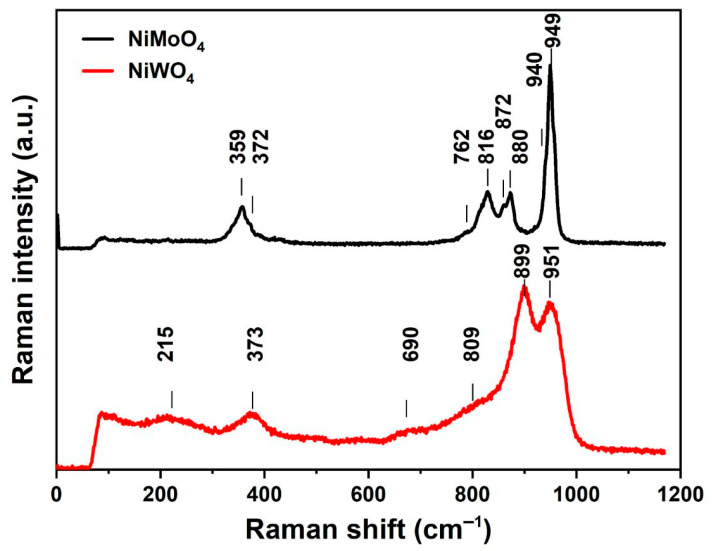
Raman spectra of hydrothermally synthesized NiMoO_4_ and NiWO_4_.

The chemical structure of the synthesized NiMoO_4_ and NiWO_4_ is characterized with Raman spectroscopy and is shown in Figure 4. The Raman spectrum for α, β-NiMoO_4_ exhibits a combination of Mo-O vibrations that are characteristics of the tetrahedral structure of the MoO_4_ anion [17,18,19] at 949,880 and 816 cm^−1^, and of peaks typical for Mo-O bonds in an octahedral coordination at 940 and 872 cm^−1^. Thereby, the bands at 949 and 940 cm^−1^ are assigned to the symmetric stretching mode of the Mo-O bond, whereas the bands observed at 880, 872, and 816 cm^−1^ are assigned to the asymmetric stretching in the O-Mo-O chain. The bands at 372 and 359 cm^−1^ are attributed to the asymmetric and symmetric bending modes of O-Mo-O, and the band at 762 cm^−1^ is associated with the symmetric stretching vibration of Ni-O-Mo [19,20,21,22]. Ross et al. [23,24] previously investigated the effect of doping metal tungstates MWO_4_ with cations of different sizes. In their studies, it was observed that the use of small metal cations (with an ionic radius < 0.77 Å) like Ni favors the formation of a distorted WO_6_ structure [23]. Doudin [25] researched further the structure of NiWO_4_, and noticed that it consists of [NiO_6_] and [WO_6_] octahedra. The octahedra are connected differently, depending on the central atom. Octahedra with the same central atom are edge-connected, whereas the octahedra with different central atoms are corner-connected [22,26]. The Ni-O bonds have similar lengths while W-O bonds are asymmetrical, leading to a distorted structure. Consequently, the Raman activity is dominated by the tungsten sub-lattice. The band at 899 cm^−1^ is assigned with the symmetric W=O stretching of the WO_6_ lattice and is in agreement with previous results [23]. The mode at 373 cm^−1^ is assigned to the symmetric vibration of NiO_6_ octahedra [27]. The bands at 809 and 690 cm^−1^ correspond to the bridging W-O-W stretching and the signal at 215 cm^−1^ to the bending W-O-W mode [23,26,28]. It should be pointed out that the band located at ~951 cm^−1^ is observed when measuring microcrystalline tungstate nanoparticles [29]. The Raman and XRD results of the synthesized materials corroborate the presence of two phases (α, β) for NiMoO_4_ and of a single phase for NiWO_4_.

**Figure 5:**
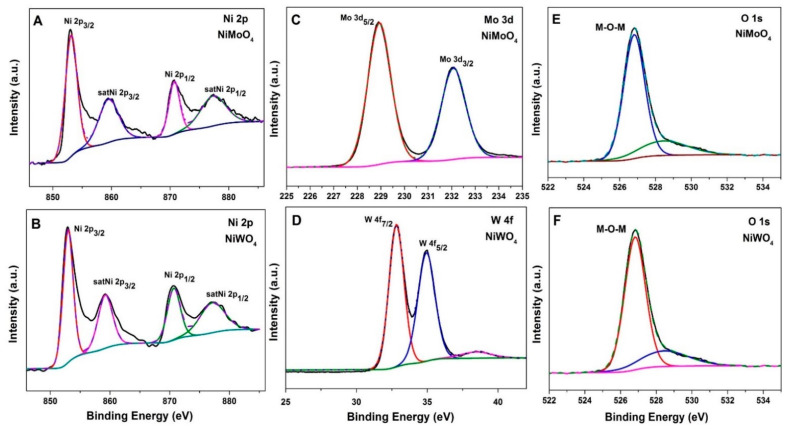
X-ray photoelectron spectra of (**A**,**B**) Ni 2p, (**C**) Mo 3d, (**D**) W 4f and (**E**,**F**) O 1s core level regions of hydrothermally synthesized NiMoO_4_ and NiWO_4_.

The chemical oxidation state of the elements in both NiMoO_4_ and NiWO_4_ were studied by X-ray photoelectron spectroscopy. The obtained spectra of each element in the as-synthesized materials are shown in Figure 5. Survey spectra of both are provided in the S1 section (Figure S2). As expected, the survey spectra show the presence of Ni 2p, Mo 3d, W 4f, and O 1s peaks in the samples. Based on the Gaussian fitting model, two major peaks with binding energy values of 853.04 and 870.91 eV could be derived for Ni 2p, which can be assigned to Ni 2p_3/2_ and Ni2p_1/2_. The energy separation of Ni 2p_3/2_–Ni 2p_1/2_ is about 17.87 eV, which indicates the presence of Ni^2+^. In addition, two satellite peaks at binding energies of 859.81 and 877.50 eV could be observed. This was observed for both NiMoO_4_ and NiWO_4_ samples, as shown in Figure 5A,B. Two major peaks at binding energy values of 228.93 and 232.05 eV for the Mo 3d spectrum were observed in the NiMoO_4_ sample (Figure 5C), relating to Mo 3d_5/2_ and Mo 3d_3/2_ levels, respectively. This spin-orbit splitting value is 3.12 eV, denoting the presence of Mo^6+^ [30]. Furthermore, the high resolution XPS spectrum of W 4f (Figure 5D) shows two major peaks with binding energies of 32.8 eV and 34.9 eV corresponding to W 4f_7/2_ and W 4f_5/2_ levels, respectively, inferring the presence of W in the +6 state. The spin energy separation of 2.1 eV is consistent with the previously reported values [31]. The typical metal oxygen metal (M-O-M) bond is observed at binding energies of 526.84 and 528.59 eV in the O 1s spectra for both NiMoO_4_ and NiWO_4_ samples (Figure 5E,F). These results confirm the formation of NiMoO_4_ and NiWO_4_.

**Figure 6:**
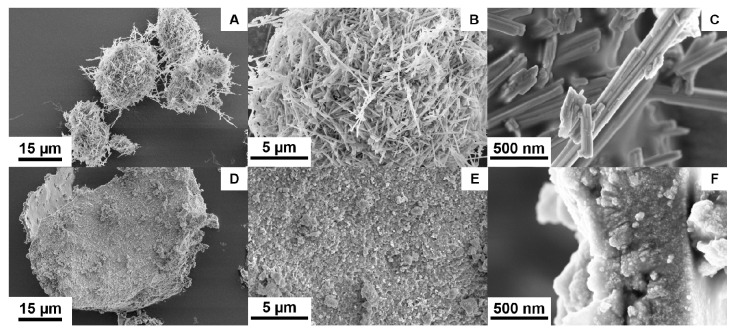
SEM images using a secondary electron (SE) detector at different magnifications: (**A**–**C**) NiMoO_4_, (**D**–**F**) NiWO_4_ samples obtained by hydrothermal synthesis.

**Figure 7:**
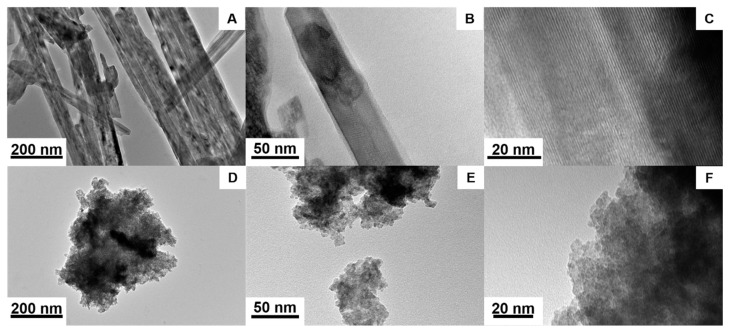
TEM images of the (**A**–**C**) NiMoO_4_ and (**D**–**F**) NiWO_4_ samples.

**Figure 8:**
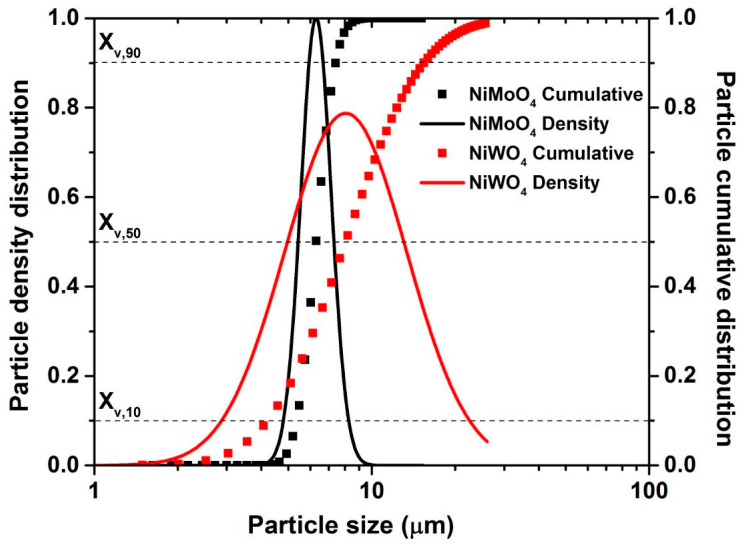
Cumulative (symbols) and density (solid lines) hydrodynamic particle size distribution obtained for NiMoO_4_ and NiWO_4_ after dispersion in N-methyl-2-pyrrolidone (NMP).

The surface topography and the particle size of the products were determined using SEM (Figure 6) and TEM images (Figure 7). The SEM images of the NiMoO_4_ sample (Figure 6A–C) show that the material consists of nanorods that are nested together into spherical agglomerates. The average hydrodynamic particle size of the agglomerates as present in N-methyl-2-pyrrolidone (NMP) dispersion is measured to be X_v,10_ = 5.2 µm, X_v,50_ = 6 µm, and X_v,90_ = 7.6 µm using sedimentation analysis (Figure 8). The individual nanorods are clearly visible in the magnified TEM images in Figure 7A–C, showing a diameter of ~40–200 nm and a length in the range of ~500 nm to 2 µm. The morphology of the NiWO_4_ particles was significantly different, with the SEM images (Figure 6D–F) showing flake-like structures with a rough surface. The average hydrodynamic size of these structures as present in NMP dispersion amounts to X_v,10_ = 3.8 µm, X_v,50_ = 8.6 µm, and X_v,90_ = 12.4 µm, which is somewhat larger than for the NiMoO_4_ sample. Figure 7D–F show that the material is composed of smaller clusters of nanoparticles. The primary nanocrystals are in the 5 nm-range, which explains the strong signal broadening in the XRD pattern. The morphological and structural variations between the two bimetallic oxides (BMO) samples have a strong impact on their respective, specific, surface area. For the NiWO_4_ sample, the specific surface area was found to be ~145 ± 7 m^2^ g^−1^, which is significantly greater than for the NiMoO_4_ system (50 ± 2 m^2^ g^−1^). The Brunauer–Emmett–Teller (BET) plots of both samples are included in the supplementary material (see Figure S3). These results suggest that the flake structures of NiWO_4_ are highly porous even though they look more compact than the NiMoO_4_ structures.

### 3.2. Electrochemical Characterization

#### 3.2.1. Three-Electrode Configuration

**Figure 9:**
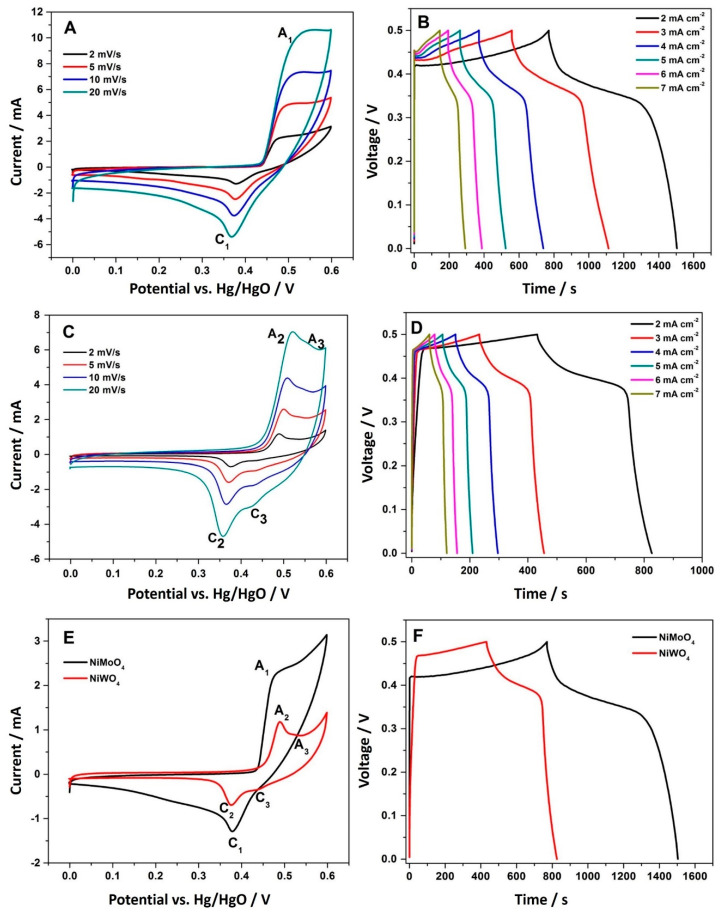
(**A**,**B**) Cyclic voltammetric (CV) curves and galvanostatic charge-discharge (CD) profiles of NiMoO_4_, (**C**,**D**) CV curves and CD profiles of NiWO_4_, (**E**,**F**) comparative CV at 2 mV/s and CD at 2 mA cm^–2^ of NiMoO_4_ and NiWO_4_, respectively. The measurements are based on a three-electrode configuration with Hg/HgO as the reference electrode.

To evaluate the role of the molybdate (MoO_4_^2−^) and tungstate (WO_4_^2−^) anion on the electrochemical properties of Ni-based bimetallic oxides (BMOs), cyclic voltammetric studies (CV) and charge-discharge (CD) measurements were carried out in 2 M NaOH aqueous solution. The active material under investigation was coated on a graphite sheet substrate as a current collector. Graphite sheet is chosen as a suitable substrate because it is highly conductive. The redox peaks observed are solely attributed to NiMoO_4_ or NiWO_4_ active material. Hence, there is no capacitance contribution from the substrate itself. The CV curves of the blank graphite electrode as well as the NiMoO_4_ and NiWO_4_ coated electrode presented in the supplementary information section (Figure S4) clarifies this. Figure 9A presents the CV curves of NiMoO_4_ at different scan rates. A pair of redox peaks (anodic A_1_, and cathodic C_1_) can be observed in a potential window of 0 to 0.6 V (vs. Hg/HgO) in all of the CV curves, implying that the redox behavior of the material is fully reversible and the charge storage in the NiMoO_4_ material is mainly governed by the electron transfer process, as given in Equation (5) [6]. The shape of the CV was retained at higher scan rates, illustrating the suitability of the material for high rate applications. The redox peak current increases linearly with an increasing sweep rate, suggesting a surface redox reaction, and the position of the redox peaks remains constant, which shows that the reaction mechanism is identical. The corresponding CD measurements at different current rates are presented in Figure 9B. The curves deviate from an ideal triangular-like shape, suggesting a characteristic pseudo-capacitive behavior. The mechanism involved in this reaction can be described as:

(5) $${\mathrm{Ni}}^{2+}{\left({\mathrm{MoO}}_{4}\right)}^{2-}+{\;\mathrm{OH}}^{-}↔{\mathrm{Ni}}^{3+}{\left({\mathrm{MoO}}_{4}\right)}^{2-}{\left(\mathrm{OH}\right)}^{-}+{\;e}^{-}\;$$

(6) $${\mathrm{Mo}}^{3+}+\;2{\mathrm{OH}}^{-}\;\to \;\mathrm{Mo}{\left(\mathrm{OH}\right)}_{2}^{\;+}$$

(7) $$\mathrm{Mo}{\left(\mathrm{OH}\right)}_{2}^{\;+}+{\mathrm{OH}}^{-}\;\to \;\mathrm{Mo}{\left(\mathrm{OH}\right)}_{3}^{}$$

(8) $$\mathrm{Mo}{\left(\mathrm{OH}\right)}_{3}^{}+5{\mathrm{OH}}^{-}\;\to {\;\mathrm{MoO}}_{4}^{\;2-}+\;4H_{2}O+\;3e^{-}$$

Similarly, Figure 9C,D present the CV and the CD curves of NiWO_4_, respectively. In comparison to the curves observed for the Mo counterpart, the shape of the CV varies, having a lower current density. On the other hand, the redox peaks are better defined, indicating that an intercalation reaction may occur during the reduction and oxidation processes. As in NiMoO_4_, a pair of redox peaks (A_1_ and C_1_) is observed at a potential window of 0 to 0.6 V (vs. Hg/HgO), showing evidence of the faradaic reaction between the Ni^2+^ and Ni^3+^ states, which is related to the diffusion of either OH^−^ ions or Na^+^ ions from the electrolyte [14]. On close inspection of the CV curves, a weak shoulder (A_3_ and C_3_) is also seen, which can be attributed to the redox behavior of tungsten in alkaline solution. In addition, CV of pristine WO_3_ in a different potential window (Figure S5) was performed. This piece of experiment helps us to elucidate the association between the anodic and cathodic peak as the two oxidation peaks coincide and are difficult to differentiate. In an alkaline solution with the presence of an excess of OH^−^ ions in the electrolyte, the molybdate reacts to Mo(OH)_3_ prior to any electrochemical processes, as shown in Equations (6)–(8), which is electrochemically inactive. The tungstate, however, is electrochemically active in an alkaline solution of pH > 8. The mechanism involved in the NiWO_4_ reaction in a solution with pH > 8 is shown in Equations (9)–(11) [32,33]. Equation (9) presents the overall mechanism taking place. Equation (10) demonstrates the reversible redox behavior of Ni, whereas Equation (11) shows, in detail, the reaction of intermediate species formed during the process.

(9) $${\mathrm{Ni}}^{2+}{\left({\mathrm{WO}}_{4}\right)}^{2-}+{\;\mathrm{OH}}^{-}\;↔{\;\mathrm{Ni}}^{3+}{\left({\mathrm{WO}}_{3}\right)}^{2-}{\left(\mathrm{OH}\right)}^{-}+{\;e}^{-}$$

(10) $${\mathrm{Ni}}^{2+}↔{\mathrm{Ni}}^{3+}+e^{-}\;\;\;\left(\mathrm{peaks}\;A1\;\mathrm{and}\;C1\right)$$

(11) $${\mathrm{WO}}_{3}+{\;\mathrm{OH}}^{-}\;\to {\mathrm{HWO}}_{4}^{-}↔WO_{4}^{\;2-}+H^{+}\;\;\left(\mathrm{peaks}\;A2\;\mathrm{and}\;C2\right)$$

Therefore, during the oxidation of NiWO_4_, WO_3_ forms in an anodic dissolution process. The degree of dissolution is directly proportional to the concentration of OH^−^ ions in the NaOH electrolyte solution [33]. This leads to the formation of a tungsten oxide layer on the surface, that passivates the electrode [32,33,34,35]. To confirm this mechanism, WO_3_ was synthesized and its electrochemical signature was verified. The CV curves for the pristine WO_3_ material (Figure S1B) exhibited an electrochemical activity at a similar potential to what is observed in Figure 9C for redox peaks A_3_ and C_3_, supporting the proposed mechanism. This behavior explains the inferior charge storage performance observed for the nickel tungstate compared to the nickel molybdate sample. In a typical case and under identical conditions, NiMoO_4_ took 1500 s to charge/discharge (Figure 9B), while the NiWO_4_ took half the amount of time (Figure 9D), resulting in a 50% loss in charge storage. Similarly, the current response (area under the curve) of the NiWO_4_ has dropped significantly. Although it is widely believed that the role of the tungstate anion is to enhance the electrical conductivity and electrochemical properties, it is not observed here. The results in Figure 9F show that the NiMoO_4_ electrode reflected its best specific capacitance at a current density of 2 mA cm^−2^. However, NiWO_4_ with its semi-crystalline form and the formation of the WO_3_ layer during the electrochemical reaction exhibited a poor specific capacitance at a current density of 2 mA cm^−2^. Therefore, the formation of WO_3_ affects the storage performance with the passivation layer further delaying the disproportionation of NiWO_4_.

#### 3.2.2. Two-Electrode Configuration

To further ascertain the performance capability of a material that could be a suitable candidate for application in pseudo-capacitors, an asymmetric device consisting of NiMoO_4_ or NiWO_4_ as the cathode and activated carbon (AC) as the anode was constructed.

**Figure 10:**
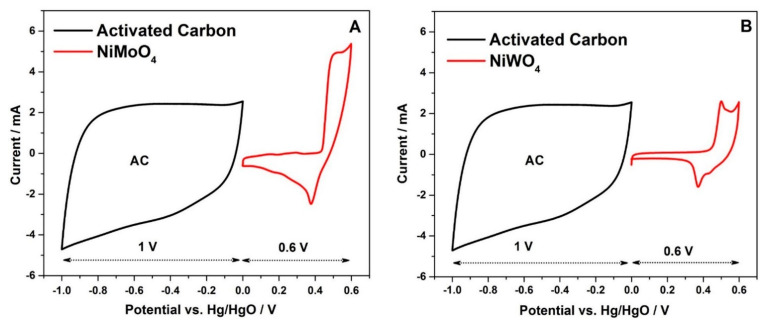
(**A**,**B**) Cyclic voltammetric curves of NiMoO_4_ and NiWO_4_ along with activated carbon (AC) recorded in a three-electrode configuration with Hg/HgO as the reference electrode.

Figure 10A,B demonstrate the CV curves recorded at a scan rate of 2 mV s^−1^ for NiMoO_4_ or NiWO_4_, AC, and Hg/HgO as the reference electrode in a three-electrode configuration. It can be inferred that NiMoO_4_ and NiWO_4_ are both suitable cathode materials, being active in the positive potential region (0 to 0.6 V) vs. Hg/HgO with activated carbon acting as the anode in the negative potential region (0 to −1 V). By coupling the electrodes together, the total potential window in a two-electrode system is, hence, determined as 1.6 V. As seen in the CV curves (see Figure 10A,B), AC shows a quasi-rectangular shape, which indicates an electric double layer mechanism for charge storage. In contrast, NiMoO_4_ and NiWO_4_ demonstrate a pair of clear redox peaks, indicative of the pseudo-capacitive nature of the material.

**Figure 11:**
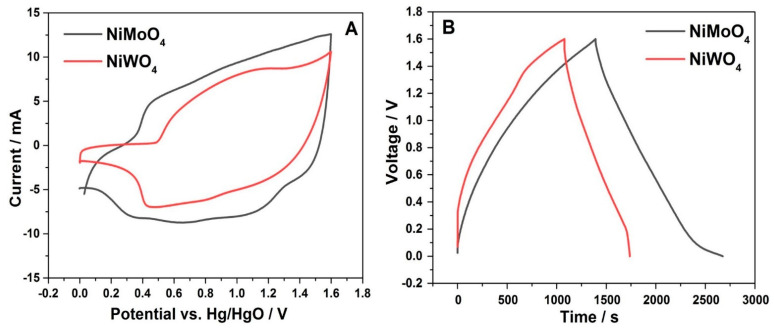
Comparative (**A**) cyclic voltammetric curves at 5 mV s^−1^, and (**B**) charge-discharge profiles at 3 mA cm^–2^ of NiMoO_4_ and NiWO_4_ asymmetric devices, respectively, in 2 M NaOH solution.

Figure 11 shows a comparative CV and CD of NiMoO_4_ and NiWO_4_ vs. AC in the asymmetric cell. The area enclosed within the CV curve of NiMoO_4_ is slightly enhanced compared to that of NiWO_4_ (Figure 11A), implying that NiMoO_4_ delivers greater specific capacitance.

This result is further supported by the corresponding charge-discharge profiles (Figure 11B), where the discharge time for the NiMoO_4_ material is longer by about 1000 s for an equal mass of the active material in both systems.

**Figure 12:**
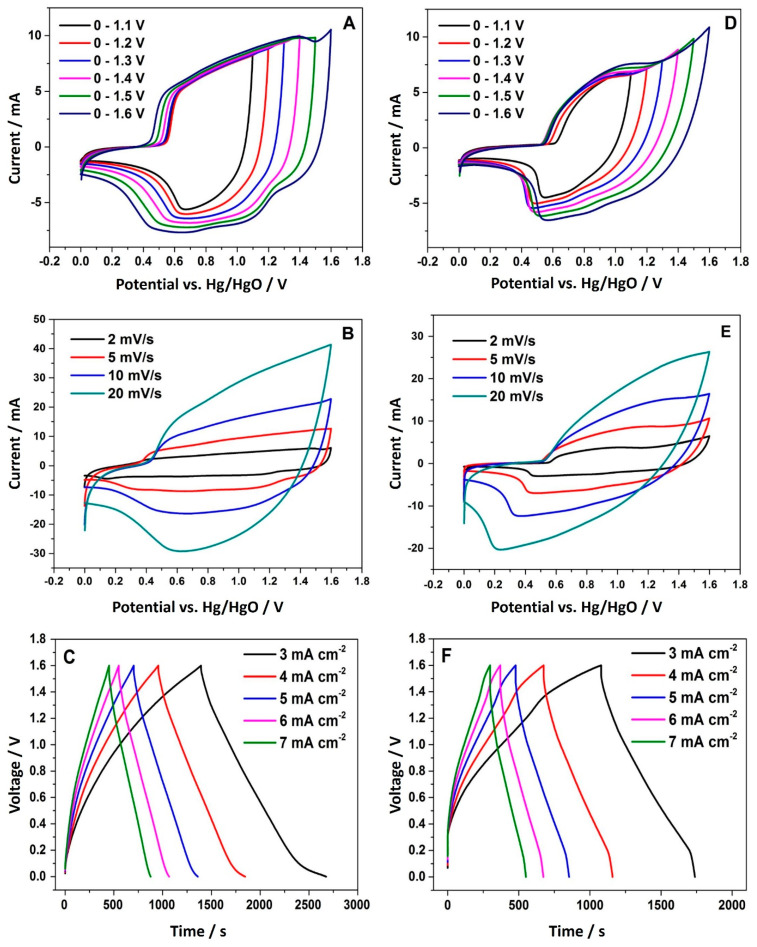
Electrochemical measurements of asymmetric cells: Cyclic voltammetric (CV) curves of NiMoO_4_ (**A**) and NiWO_4_ (**D**) at different potential windows at a scan rate of 5 mV s^−1^, CV curves of NiMoO_4_ (**B**), and NiWO_4_ (**E**) at different scan rates and galvanostatic charge-discharge (CD) profile of NiMoO_4_ (**C**) and NiWO_4_ (**F**) at different applied currents.

A detailed electrochemical investigation of NiMoO_4_ and NiWO_4_ is shown in Figure 12. Using Equation (1), the specific capacitance at an applied current of 3 mA cm^−2^ was found to be 134 and 83 F·g^−1^ for NiMoO_4_ and NiWO_4_, respectively. Both materials were able to retain a high specific capacitance of 82.87 F·g^−1^ (NiMoO_4_) and 74.37 F·g^−1^ (NiWO_4_) even at a higher applied current of 7 mA cm^−2^. Using Equations (2) and (3), the energy and power density at 3 mA cm^−2^ applied current were calculated to be 47.52 W h kg^−1^ and 481.11 W kg^−1^ for NiMoO_4_, and 29.4 W h kg^−1^ and 575.56 W kg^−1^ for NiWO_4_, respectively. A combination of redox and electric double layer capacitator (EDLC) behavior was observed in the CV curves (Figure 12A,D), which illustrates the pseudocapacitive nature of NiMoO_4_ and NiWO_4_ and the electric double layer property of AC.

**Figure 13:**
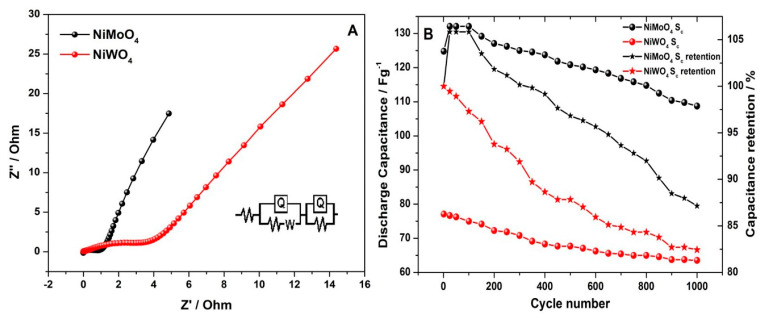
(**A**) Electrochemical impedance spectroscopy (EIS) results of NiMoO_4_ and NiWO_4_ hybrid cells presented as a comparative Nyquist plot and (**B**) cycle stability at an applied current density of 5 mA cm^–2^ demonstrating the device capacitance and its retention percentage.

To obtain further insights on the charge transport behavior of both materials, electrochemical impedance spectroscopy (EIS) was performed (Figure 13A). The fitted equivalent circuit for both Nyquist plots is shown in the inset. Each plot shows two distinct regions: a semi-circle in the high-frequency area corresponds to R_ct_ (charge transfer resistance) and R_s_ (solution resistance) of the hybrid cell, followed by a linear region in the low-frequency area, which relates to chemical diffusion impedance, termed as Warburg impedance (W). It is noted that the diameter of the semicircle arc in the high frequency region is larger for NiWO_4_. This represents the difference in electrochemical charge transport between NiMoO_4_ and NiWO_4_, with a supercapacitor-type and hybrid-type behavior, respectively. The values of R_ct_, R_s_, and W for NiMoO_4_ vs. AC were calculated to be 0.019 Ω cm^2^, 1.04 Ω cm^2^, and 3.175 Ω cm^2^ s^−1/2^. In the case of NiWO_4_ vs. AC, the values were found to be 0.284 Ω cm^2^, 3.712 Ω cm^2^, and 1.466 Ω cm^2^ s^−1/2^ for R_ct_, R_s_, and W, respectively. The steep slope of the Warburg line for NiMoO_4_ relates to the good diffusion capability of ions in the system. Furthermore, the surface film resistance values (R_f_) for NiMoO_4_ vs. AC and NiWO_4_ vs. AC were found to be 18.7 Ω cm^2^ and 32.8 Ω cm^2^, respectively, which corresponds to a solid electrolyte interface (SEI) layer formation. An additional constant phase element (CPE), denoted as Q in the circuit, represents the non-ideal capacitive behavior of the electrode material. This parameter originates from redox mechanisms being involved during the energy storage process. The fitted results showed CPE values of 1.42 × 10^−3^ Ω^−1^ cm^2^ s and 2.35 × 10^−3^ Ω^−1^ cm^2^ s for the NiMoO_4_ vs. AC device, while 3.28 × 10^−3^ Ω^−1^ cm^2^ s and 4.12 × 10^−3^ Ω^−1^ cm^2^ s were obtained for NiWO_4_ vs. AC. This data supports the overall superior electrochemical performance of the NiMoO_4_ electrode. Arguably, if the tungstate anion were beneficial for enhancing the electrical conductivity, the observed impedance values should be lower than for the molybdate. Therefore, the role of the anodic dissolution of WO_3_ plays a dominant role, determining the electrochemical performance rather than the conductivity. The result shows that the electrolyte ions and electrons show higher transfer rates in NiMoO_4_.

For the practicality in applications, electrochemical stability is an important factor to be considered for pseudo-capacitors and it is presented in Figure 13B. The charge-discharge measurements were performed within a voltage window of 1.6 V at a current density of 5 mA cm^−2^ for 1000 continuous cycles. It is observed that, at the end of the investigation, the asymmetric devices, NiMoO_4_ vs. AC and NiWO_4_ vs. AC retained 87.14% and 82.44% of their initial capacitance, respectively. The relative difference in capacity loss between the two materials is marginal. However, their initial storage capacitance values are significantly different. This is attributed to the role of the anions in the binary metal oxide electrodes. Thus, NiMoO_4_ is highly promising as potential electrode material, while the anodic dissolution of WO_3_ in alkaline solution passivates the NiWO_4_ electrode, which is reducing its storage capacitance.

## 4. Conclusions

NiMoO_4_ and NiWO_4_ as two Ni-based BMOs were synthesized via a hydrothermal method and electrochemically tested as hybrid electrodes for pseudo-capacitors. The characterization of the samples via XRD, XPS, and Raman corroborates that both synthesized materials are a combination of Ni-Mo and Ni-W in the predicted atomic ratio forming a monoclinic crystal structure. The morphology and specific surface area of the prepared materials were significantly different, with NiWO_4_ exhibiting a high specific surface area, which is usually considered advantageous to increasing the specific capacitance. However, NiMoO_4_ delivers better electrochemical properties, suggesting that the redox reaction mechanism has a higher influence than the morphology. The redox reaction for NiMoO_4_ only involves the faradaic reaction between Ni^2+^ and Ni^3+^ at the surface, allowing high capacity and longer charge storage performance. NiWO_4_ shows a two-step redox reaction with, on one hand, the contribution from Ni and, on the other hand, the participation of a tungsten oxide layer. The latter is the result of a reaction of tungstate with the electrolyte, forming WO_3_, which is deposited at the surface of the electrode, forming a passivation layer that decreases the capacitance. As the main conclusion, NiMoO_4_ should be considered as the favorite candidate for pseudo-capacitor applications. The anion of the compound, hence, can play a decisive role in BMOs for electrochemical applications and lead to substantial changes in the charge storage mechanism.

## Data Availability

The data presented in this study are available within this article. Further inquiries may be directed to the authors.

## Supplementary Materials

The following are available online at https://www.mdpi.com/2079-4991/11/3/580/s1. S1. Hydrothermal synthesis of tungsten oxide (WO_3_). Figure S1: (**A**) X-ray diffraction (XRD) pattern of hydrothermally synthesized WO_3_ and (**B**) cyclic voltammetric (CV) curves of WO_3_ with different scan rates in 2 M NaOH solution based on three-electrode configuration with Hg/HgO as the reference electrode. Figure S2: XPS survey spectra of NiMoO_4_ (black color) and NiWO_4_ (red color). Figure S3: BET plot of NiMoO_4_ and NiWO_4_ using points collected at the pressure range between 0.1 to 0.3. Figure S4: A comparative cyclic voltammetric (CV) curves of a blank graphite sheet. NiMoO_4_-coated and NiWO_4_-coated graphite sheet to demonstrate no capacitance contribution is coming from the graphite substrate. The experiment is carried out in three-electrode configurations with Hg/HgO as the reference electrode at 2 mV/s scan rate in 2 M NaOH solution. Figure S5: Cyclic voltammetric (CV) curves of WO_3_ at 5 mV/s in 2 M NaOH solution based on three-electrode configuration with Hg/HgO as the reference electrode with a different potential window. This experiment helps us to elucidate the association between an anodic and cathodic peak as the two oxidation peaks coincide and are difficult to differentiate.

## References

[1] Miller E.E., Hua Y., Tezel F.H. (2018). Materials for energy storage: Review of electrode materials and methods of increasing capacitance for supercapacitors. J. Energy Storage.

[2] An C., Zhang Y., Guo H., Wang Y. (2019). Metal oxide-based supercapacitors: Progress and prospectives. Nanoscale Adv..

[3] Minakshi M., Mitchell D.R.G., Jones R.T., Pramanik N.C., Jean-Fulcrand A., Garnweitner G. (2020). A Hybrid Electrochemical Energy Storage Device Using Sustainable Electrode Materials. ChemistrySelect.

[4] Wang G., Zhang L., Zhang J. (2012). A review of electrode materials for electrochemical supercapacitors. Chem. Soc. Rev..

[5] Zhang G., Lou X.W.D. (2013). General solution growth of mesoporous NiCo_2_O_4_ nanosheets on various conductive substrates as high-performance electrodes for supercapacitors. Adv. Mater..

[6] Cai D., Wang D., Liu B., Wang Y., Liu Y., Wang L., Li H., Huang H., Li Q., Wang T. (2013). Comparison of the electrochemical performance of NiMoO_4_ nanorods and hierarchical nanospheres for supercapacitor applications. ACS Appl. Mater. Interfaces.

[7] Tang X., Zhang B., Lui Y.H., Hu S. (2019). Ni-Mn bimetallic oxide nanosheets as high-performance electrode materials for asymmetric supercapacitors. J. Energy Storage.

[8] De Oliveira A.L.M., Ferreira J.M., Silva M.R.S., de Souza S.C., Vieira F.T.G., Longo E., Souza A.G., Santos I.M.G. (2009). Influence of the thermal treatment in the crystallization of NiWO_4_ and ZnWO_4_. J. Therm. Anal. Calorim..

[9] Baoyi S., Aiju X., Jiang W. (2016). The impact of preparation methods on the structure and catalytic performance of NiMoO_4_ for oxidative dehydrogenation of propane. Integr. Ferroelectr..

[10] Klissurski D., Mancheva M., Iordanova R., Tyuliev G., Kunev B. (2006). Mechanochemical synthesis of nanocrystalline nickel molybdates. J. Alloys Compd..

[11] AlShehri S.M., Ahmed J., Alzahrani A.M., Ahamad T. (2017). Synthesis, characterization, and enhanced photocatalytic properties of NiWO_4_ nanobricks. New J. Chem..

[12] Wang B., Li S., Wu X., Tian W., Liu J., Yu M. (2015). Integration of network-like porous NiMoO_4_ nanoarchitectures assembled with ultrathin mesoporous nanosheets on three-dimensional graphene foam for highly reversible lithium storage. J. Mater. Chem. A.

[13] Moreno B., Chinarro E., Colomer M.T., Jurado J.R. (2010). Combustion Synthesis and Electrical Behavior of Nanometric β-NiMoO_4_. J. Phys. Chem. C.

[14] Niu L., Li Z., Xu Y., Sun J., Hong W., Liu X., Wang J., Yang S. (2013). Simple synthesis of amorphous NiWO_4_ nanostructure and its application as a novel cathode material for asymmetric supercapacitors. ACS Appl. Mater. Interfaces.

[15] Chen S., Yang G., Jia Y., Zheng H. (2017). Three-dimensional NiCo_2_O_4_@NiWO_4_ core–shell nanowire arrays for high performance supercapacitors. J. Mater. Chem. A.

[16] Garnweitner G., Xue D. (2020). Crystal engineering for electrochemical applications. CrystEngComm.

[17] Chen Y.-Y., Zhang Y., Zhang X., Tang T., Luo H., Niu S., Dai Z.-H., Wan L.-J., Hu J.-S. (2017). Self-Templated Fabrication of MoNi4 /MoO3-x Nanorod Arrays with Dual Active Components for Highly Efficient Hydrogen Evolution. Adv. Mater..

[18] Dury F., Gaigneaux E.M., Ruiz P. (2003). The active role of CO2 at low temperature in oxidation processes: The case of the oxidative dehydrogenation of propane on NiMoO4 catalysts. Appl. Catal. A.

[19] De Moura A.P., de Oliveira L.H., Rosa I.L.V., Xavier C.S., Lisboa-Filho P.N., Li M.S., La Porta F.A., Longo E., Varela J.A. (2015). Structural, optical, and magnetic properties of NiMoO_4_ nanorods prepared by microwave sintering. Sci. World J..

[20] Abdel-Dayem H.M. (2007). Dynamic Phenomena during Reduction of α-NiMoO_4_ in Different Atmospheres: In-Situ Thermo-Raman Spectroscopy Study. Ind. Eng. Chem. Res..

[21] Zou J.Y., Schrader G.L. (1997). Deposition of multiphase molybdate thin films by reactive sputtering. Thin Solid Films.

[22] Hanuza J., Maczka M., Van der Maas J.H. (1995). Vibrational Properties of Double Tungstates of the MIMIII(WO4)2 Family (MI = Li, Na, K; MIII = Bi, Cr). J. Solid State Chem..

[23] Ross-Medgaarden E.I., Wachs I.E. (2007). Structural Determination of Bulk and Surface Tungsten Oxides with UV−vis Diffuse Reflectance Spectroscopy and Raman Spectroscopy. J. Phys. Chem. C.

[24] Zawawi S.M., Yahya R., Hassan A., Ekramul Mahmud H.N.M., Daud M.N. (2013). Structural and optical characterization of metal tungstates (MWO4; M=Ni, Ba, Bi) synthesized by a sucrose-templated method. Chem. Cent. J..

[25] Doudin N., Pomp S., Blatnik M., Resel R., Vorokhta M., Goniakowski J., Noguera C., Netzer F.P., Surnev S. (2017). Epitaxial NiWO4 films on Ni(110): Experimental and theoretical study of surface stability. Surface Sci..

[26] Vroulias D., Gkoulemani N., Papadopoulou C., Matralis H. (2019). W–modified Ni/Al_2_O_3_ catalysts for the dry reforming of methane: Effect of W loading. Catal. Today.

[27] Lima N.A., Alencar L.D.S., Siu-Li M., Feitosa C.A.C., Mesquita A., M’peko J.-C., Bernardi M.I.B. (2020). NiWO4 powders prepared via polymeric precursor method for application as ceramic luminescent pigments. J. Adv. Ceram..

[28] Harshan H., Priyanka K.P., Sreedevi A., Jose A., Varghese T. (2018). Structural, optical and magnetic properties of nanophase NiWO4 for potential applications. Eur. Phys. J. B.

[29] Anspoks A., Kalinko A., Timoshenko J., Kuzmin A. (2014). Local structure relaxation in nanosized tungstates. Solid State Commun..

[30] Sharma P., Minakshi Sundaram M., Watcharatharapong T., Laird D., Euchner H., Ahuja R. (2020). Zn Metal Atom Doping on the Surface Plane of One-Dimesional NiMoO4 Nanorods with Improved Redox Chemistry. ACS Appl. Mater. Interfaces.

[31] Green S.V., Kuzmin A., Purans J., Granqvist C.G., Niklasson G.A. (2011). Structure and composition of sputter-deposited nickel-tungsten oxide films. Thin Solid Films.

[32] Heumann T.H., Stolica N. (1971). The electrochemical behaviour of tungsten-II. The dissolution of tungsten in NaOH solutions. Electrochim. Acta.

[33] Tuvić T., Pašti I., Mentus S. (2011). Tungsten electrochemistry in alkaline solutions—Anodic dissolution and oxygen reduction reaction. Russ. J. Phys. Chem..

[34] Ortiz P.I., Giordano M.C., Lopez Teijelo M. (1988). Electrochemical behaviour of tungsten in alkaline media Part II. Sodium carbonate solutions. J. Electroanal. Chem. Interfacial Electrochem..

[35] Krtil P., Fattakhova D., Yoshimura M. (2002). Mechanism of soft solution processing formation of alkaline earth metal tungstates: An electrochemical and in situ AFM study. J. Solid State Electrochem..

