# Supplementary material for: Effect of the Anionic Counterpart: Molybdate vs. Tungstate in Energy Storage for Pseudo-Capacitor Applications
Source: Nanomaterials (Basel). 2021 Feb 26;11(3):580. doi: 10.3390/nano11030580 (PMC7996838; doi:10.3390/nano11030580)
Supplement: Supplementary file 1 [file nanomaterials-11-00580-s001.pdf]

nanomaterials

Supporting Information

Effect of the Anionic Counterpart: Molybdate vs. Tungstate in Energy Storage for a Pseudo-Capacitor Application

Pratigya Sharma [1], Manickam Minakshi [1,*], Jonathan Whale [1], Annelise Jean-Fulcrand [2,3] and Georg Garnweitner [2,3,*]

[1] Engineering and Energy, Murdoch University, WA 6150, Australia; Pratigya.Sharma@murdoch.edu.au (P.S.); j.whale@murdoch.edu.au (J.W.)
[2] Technische Universität Braunschweig, Institut für Partikeltechnik, Volkmaroder Straße 5, 38104 Braunschweig, Germany; a.jean-fulcrand@tu-braunschweig.de
[3] Technische Universität Braunschweig, Laboratory for Emerging Nanometrology, Langer Kamp 6A, 38106 Braunschweig, Germany;
* Correspondence: minakshi@murdoch.edu.au (M. M.); g.garnweitner@tu-braunschweig.de (G. G.)

S1. Hydrothermal Synthesis of Tungsten Oxide (WO_3)

For the synthesis of WO_3, 1 mmol of sodium tungstate was dissolved well under sonication in deionized water. To the above solution, HCl solution was added drop wise and mixed thoroughly. The homogeneous solution was then transferred to a 50 mL Teflon-lined sealed autoclave. The reaction was then carried out at 140 °C for 12 h. The obtained precipitate was washed with ethanol and deionised water and finally dried overnight in an oven maintained at 60 °C.

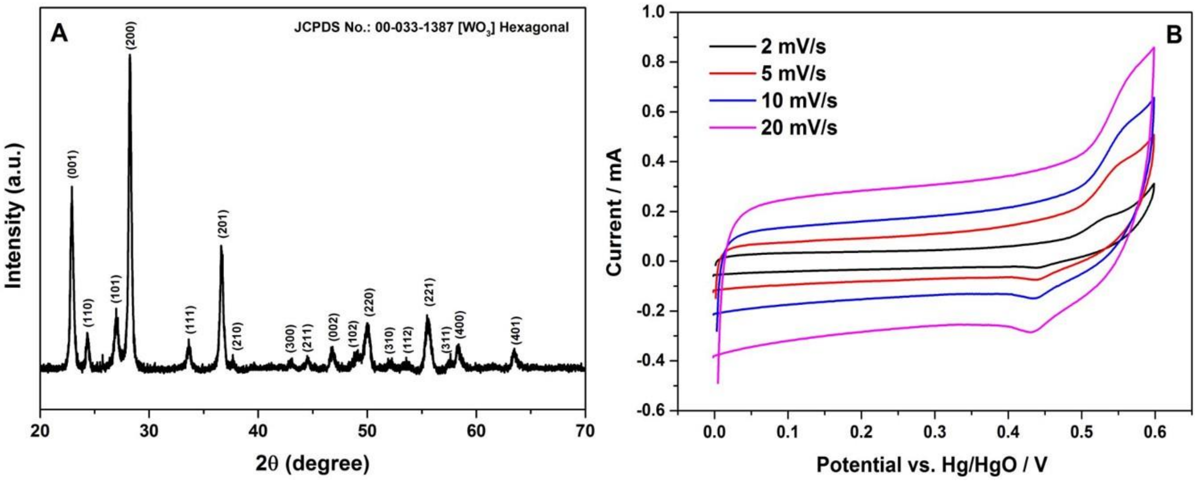

Figure S1. (**A**) X-ray diffraction (XRD) pattern of hydrothermally synthesized WO_3 and (**B**) Cyclic voltammetric (CV) curves of WO_3 with different scan rates in 2 M NaOH solution based on three-electrode configuration with Hg/HgO as the reference electrode.

Figure. S1 A shows the XRD pattern of as-synthesized product. The pattern well matches with the hexagonal phase of WO_3 (JCPDS No. 33-1387) with the lattice constant values as a = 7.298 Å, b = 7.29 Å and c = 3.899 Å. Hence, we confirm the presence of h-WO_3 with no other impurities or phases being detected.

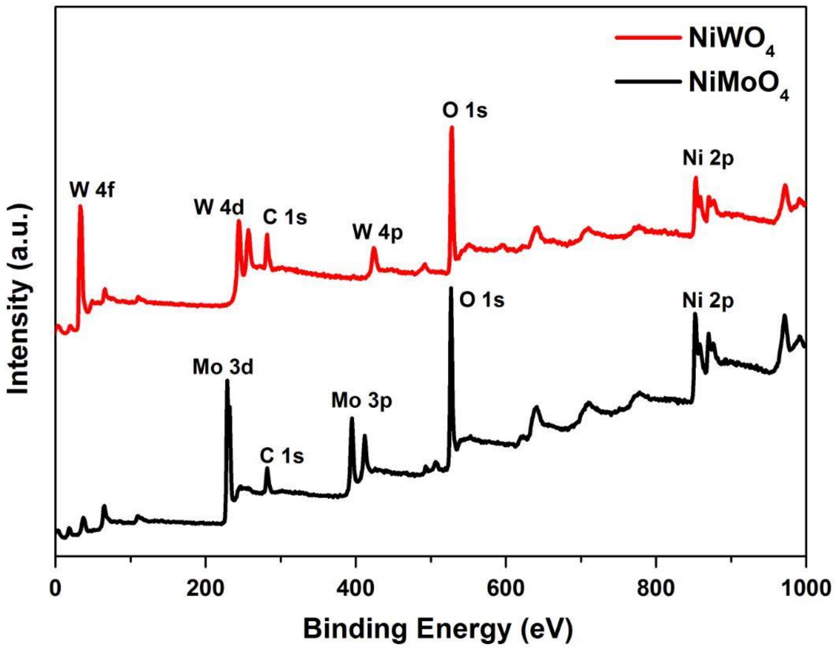

Figure S2. XPS survey spectra of $NiMoO_4$ (black color) and $NiWO_4$ (red color).

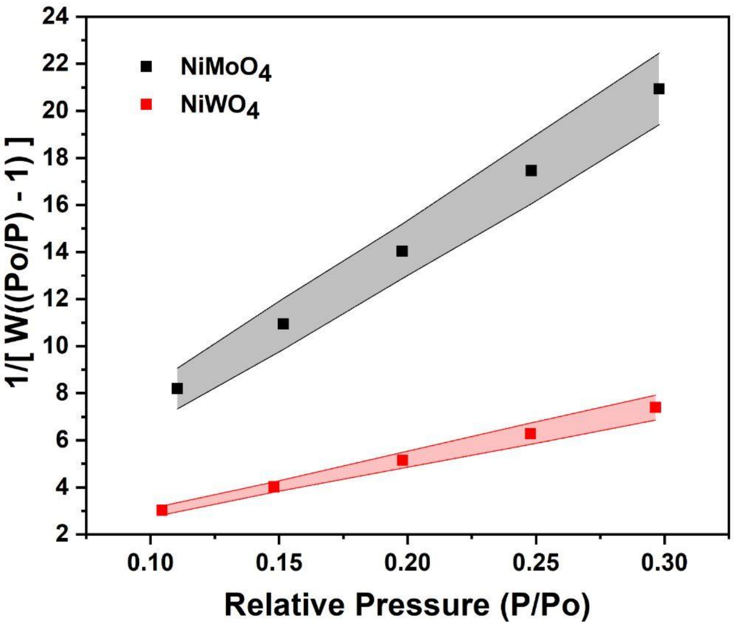

Figure S3. BET plot of $NiMoO_4$ and $NiWO_4$ using points collected at the pressure range 0.1 to 0.3.

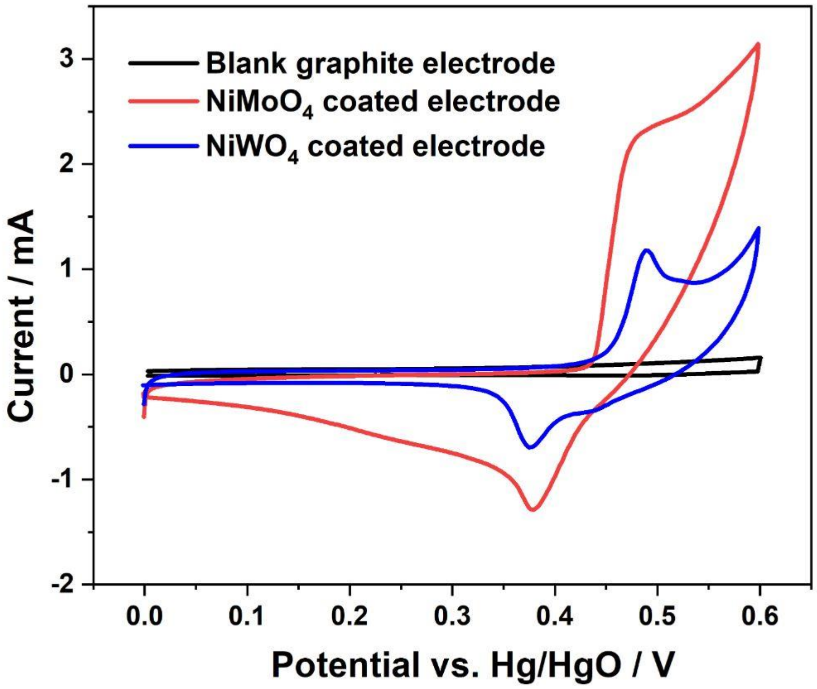

Figure S4. Cyclic voltammetric (CV) profile of $NiMoO_4$ and $NiWO_4$ coated on a graphite sheet. A blank graphite electrode has been compared under identical conditions to demonstrate there is no capacitance contribution. The experiment is carried out in three electrode configuration with Hg/HgO as the reference electrode at a scan rate of 2 mV/s in a 2 M NaOH solution.

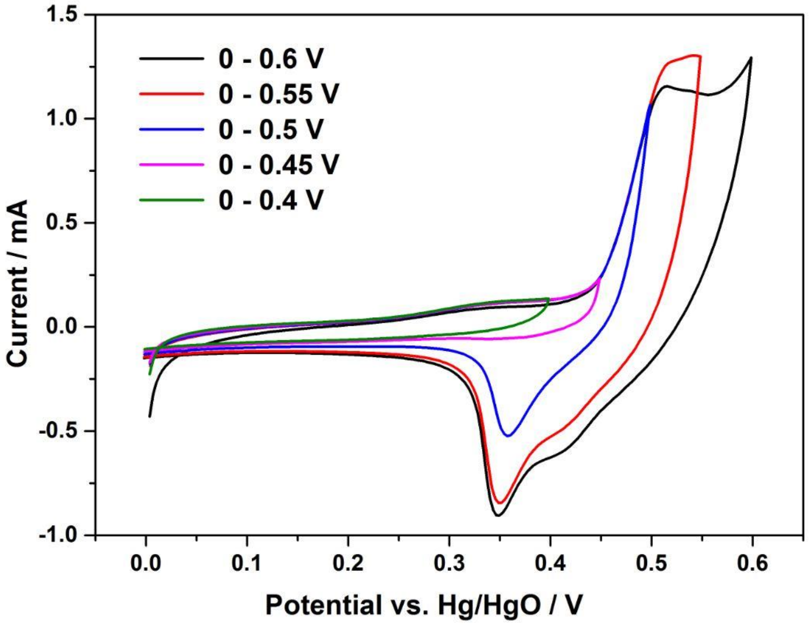

Figure S5. Cyclic voltammetric (CV) curves of WO_3 at 5mV/s in 2 M NaOH solution based on three-electrode configuration with Hg/HgO as the reference electrode with different potential window. This experiment helps us to elucidate the association between anodic and cathodic peak as the two oxidation peaks coincide and are difficult to differentiate.
